# Left Atrioventricular Coupling Index: An Echocardiographic Index of Atrioventricular Dysfunction in Dogs with Myxomatous Mitral Valve Disease

**DOI:** 10.3390/vetsci13020201

**Published:** 2026-02-20

**Authors:** Federica Valeri, Francesco Porciello, Mark Rishniw, Simone Cupido, Maria Cicogna, Andrea Corda, Domenico Caivano

**Affiliations:** 1 Department of Veterinary Medicine, University of Perugia, 06126 Perugia, Italy; federica.valeri@dottorandi.unipg.it (F.V.); francesco.porciello@unipg.it (F.P.);; 2 Veterinary Information Network, Davis, CA 95616, USA; mr89@cornell.edu; 3 Department of Veterinary Medicine, University of Sassari, 07100 Sassari, Italy; acorda1@uniss.it

**Keywords:** mitral valve disease, left atrium, left ventricle, echocardiography, canine

## Abstract

The close physiological relationship between the left atrium (LA) and left ventricle (LV) suggests that an index assessing both the cardiac chambers simultaneously could provide useful information about disease severity. Consequently, investigators have proposed the atrioventricular coupling index (LACi) and demonstrated its utility in predicting the likelihood of atrial fibrillation, heart failure, and other cardiovascular events in humans. No studies have been reported in veterinary medicine. Therefore, we measured the LACi in healthy dogs and dogs affected by myxomatous mitral valve disease (MMVD). Two hundred and thirty-three dogs (105 healthy dogs and 128 dogs with MMVD) were retrospectively included in the study. The LACi (LA volume/LV volume × 100) at LV end-diastole (LACi-ED) and LV end-systole (LACi-ES) of each dog was measured using a monoplane Simpson’s Method of Discs from the left apical four-chamber view. In healthy dogs, LACi-ED and LACi-ES showed no relationship with bodyweight, heart rate and age (R^2^ < 0.03, for all variables). In MMVD dogs, LACi-ED and LACi-ES differed between ACVIM stages (*p* < 0.001 and *p* < 0.02, for all stages). The LACi-ED and LACi-ES had similar accuracy in identifying MMVD dogs with congestive heart failure (area under the curve of 0.920 and 0.906, respectively). Our data suggest that the LACi can be useful in assessing left atrioventricular function in dogs with MMVD but the diagnostic accuracy in identifying dogs with congestive heart failure was not superior to the left atrial-to-aortic ratio. Prospective studies are needed to evaluate the predictive value of this new echocardiographic index in dogs affected by MMVD.

## Simple Summary

Given the close physiological relationship between the left atrium (LA) and left ventricle (LV), an index assessing both chambers simultaneously could be useful for evaluating disease severity. Therefore, we measured the left atrioventricular coupling index (LACi) in healthy dogs and dogs affected by myxomatous mitral valve disease (MMVD). The LACi (LA volume/LV volume*100) at LV end-diastole (LACi-ED) and LV end-systole (LACi-ES) were retrospectively measured in 233 dogs (105 healthy dogs and 128 dogs with MMVD). LACi-ED and LACi-ES differed between ACVIM stages (*p* < 0.001 and *p* < 0.02, for all stages) and showed similar accuracy to each other in identifying MMVD dogs with congestive heart failure. Our data suggest that the LACi can be useful in assessing left atrioventricular function in dogs with MMVD, but the diagnostic accuracy in identifying dogs with congestive heart failure is similar to traditional echocardiographic indices. Prospective studies are needed to evaluate the predictive value of this new echocardiographic index in dogs affected by MMVD.

## 1. Introduction

Myxomatous mitral valve disease (MMVD) is the most commonly acquired cardiac condition in adult and geriatric dogs, particularly small- to medium-sized dogs [1]. MMVD is mostly a chronic, slowly progressive disorder characterized by myxomatous degeneration of the mitral valve. The progression of the disease results in increased left atrioventricular volume overload, atrial dilation, ventricular eccentric hypertrophy and can lead to congestive heart failure (CHF). Echocardiography plays a crucial role in the diagnosis and staging of MMVD, using reliable markers to identify atrioventricular enlargement such as the left atrial-to-aortic ratio (LA:Ao) and left internal diameter in the diastole normalized to body weight (LVIDDn) [1,2,3]. However, these parameters assess the chambers independently.

The close relationship between the left atrium (LA) and the left ventricle (LV) is critical for efficient cardiac function. This interplay suggests that the assessment of both cardiac chambers could better reflect atrioventricular dysfunction and can be used as a useful index for heart failure. The left atrioventricular coupling index (LACi), defined by the ratio between LA and LV end-diastolic volume, has emerged as a novel echocardiographic index in human medicine. This index has shown promising results in risk stratification, in predicting cardiovascular disease progression and as an early marker of cardiovascular dysfunction in conditions such as atrial fibrillation and heart failure [4,5,6,7].

No studies have assessed the LACi in veterinary medicine. Therefore, the aim of this study was to provide reference intervals for the LACi, and to investigate the association between the LACi and the severity of MMVD compared with the current ACVIM grading system. We assessed the LACi at both the end-diastole and end-systole to explore atrioventricular coupling during two distinct phases of the cardiac cycle, which can be differentially affected in MMVD.

## 2. Materials and Methods

### 2.1. Data Collection

This retrospective study included 233 adult dogs (>1 year) selected from an ongoing database of the University Teaching Hospital of Perugia from January 2017 to February 2025: 105 healthy dogs and 128 affected by MMVD [B1 stage (n = 38), B2 (n = 52) and C (n = 38)]. Dogs with persistent non-sinus rhythms or inadequate image quality for left apical four-chamber view were excluded. No dogs were excluded based on breed, age, sex and bodyweight. All healthy dogs had been recruited for prior echocardiographic studies and had no history of disease or abnormal findings at physical examination. Dogs affected with MMVD were staged according to the ACVIM guidelines [1].

**Figure 1:**
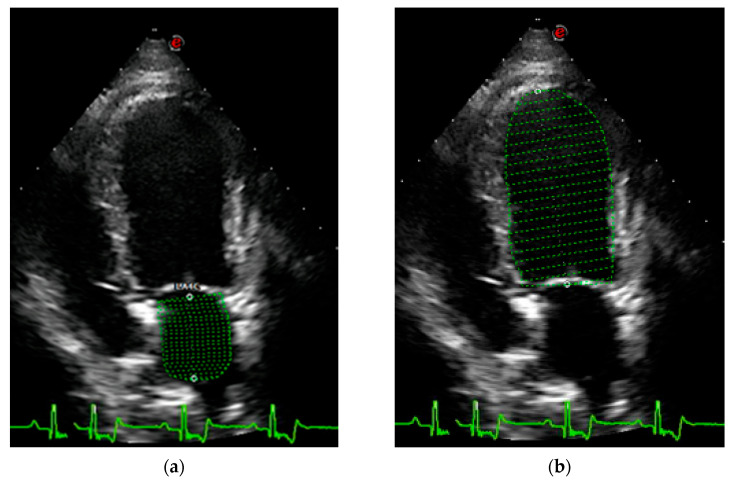
Representative echocardiographic images of view and method of obtaining left atrial and left ventricle volume estimates using Simpson’s Method of Discs (green lines). Left atrial (**a**) and left ventricle (**b**) volumes estimates obtained from left apical four-chamber view at end-diastole.

The same investigator (FV) selected appropriate frames from stored cineloops of each dog and performed the measurements from those frames. Left atrial and LV volumes were measured at the same end-diastolic phase defined by the frame just after mitral valve closure (around the onset of the QRS complex) and at the same end systolic phase defined by the frame just before mitral valve opening (typically after the end of the T wave). Echocardiographic volumes were measured using a single-plane Simpson’s Method of Discs from the apical four-chamber view. The internal border of the LA was traced manually, beginning at the septal mitral annulus (first hinge point), extending around the LA roof and ending at the lateral mitral annulus (second hinge point), using the blood–tissue interface (Figure 1). The ostia of the pulmonary veins were excluded, and a straight line drawn from hinge point to hinge point across the mitral valve annulus defined the boundary of the LA. The height of the stacked discs was selected to be perpendicular to the midpoint of the mitral valve annulus, bisecting the LA. The internal border of the LV was traced manually, beginning at the septal mitral annulus, extending around the apex and ending at the lateral mitral annulus, using the blood–tissue interface (Figure 1). These two points were joined by a straight line by software on the ultrasound system. The maximum length of the LV was defined by the distance between the mitral annulus and the apex.

Left atrial and ventricular volume measurements were used to calculate the LACi at the end-diastole (LACi-ED) and LACi at the end-systole (LACi-ES), calculated using the following:

- LACi-ED (%) = LA end-diastolic volume (LAEDV)/LV end-diastolic volume (LVEDV) × 100.
- LACi-ES (%) = LA end-systolic volume (LAESV)/LV end-systolic volume (LVESV) × 100.

The sum of the LA:Ao and LVIDDn (LA:Ao + LVIDDn) was also calculated, as a linear combined severity score [8,9]. All measurements were performed three times, and the average of the three values was used for analysis.

### 2.2. Statistical Analysis

The relationships between body weight, heart rate, and age and the LACi-ES and LACi-ED in healthy dogs were examined by creating scatter plots and performing univariable linear regression analyses where appropriate. Reference intervals (and 90% confidence intervals around the reference limits) were generated from healthy dogs using Reference Value Advisor software [10]. A non-parametric method was used, as this approach was identified by the software as appropriate for the provided data.

Differences in the LACi-ED and LACi-ES across ACVIM stages (Healthy, B1, B2, and C) were assessed using a Kruskal–Wallis test followed by pairwise comparisons with the Dunn–Conover method. These indices were also compared within the ACVIM B stage after stratifying dogs into “mild”, “moderate”, and “severe” subclinical disease categories, according to a previously proposed classification scheme [11], using either Kruskal–Wallis tests with post hoc pairwise comparisons or Mann–Whitney U tests (when only two disease severity levels were compared). The distributions of LACi-ED and LACi-ES values across disease stages were visually represented using box plots.

The diagnostic utility of LACi variables, LA:Ao, and LA:Ao + LVIDDn for identifying dogs with CHF was evaluated using receiver operating characteristic (ROC) curve analysis. Selected ROC curves were compared to determine if any variables were substantially superior to others in the diagnosis of CHF. For this analysis, only dogs in ACVIM stage B2 or those with CHF were included (n = 90).

## 3. Results

The study sample consisted of 233 dogs, including of 105 healthy and 128 affected by MMVD. Healthy dogs ranged in bodyweight from 3.8 to 45 kg (median: 17 kg) and were aged between 1 and 14 years (median: 4 years). MMVD dogs ranged in bodyweight from 2.5 to 32.2 kg (median: 9.6 kg) and were aged between 3 and 17 years (median: 11 years) (Table 1). Thirty-eight dogs were classified as ACVIM stage B1, fifty-two as ACVIM stage B2 and thirty-eight had CHF (ACVIM stage C).

A moderate relationship was observed between the LACi-ED and mitral E peak velocity (R^2^ = 0.55), whereas a weak relationship was found between the LACi-ED and the mitral E/A peak ratio (R^2^ = 0.20). No relationship was detected between the LACi-ES and either mitral E peak velocity or the mitral E/A ratio (R^2^ < 0.03, for both).

**Figure 2:**
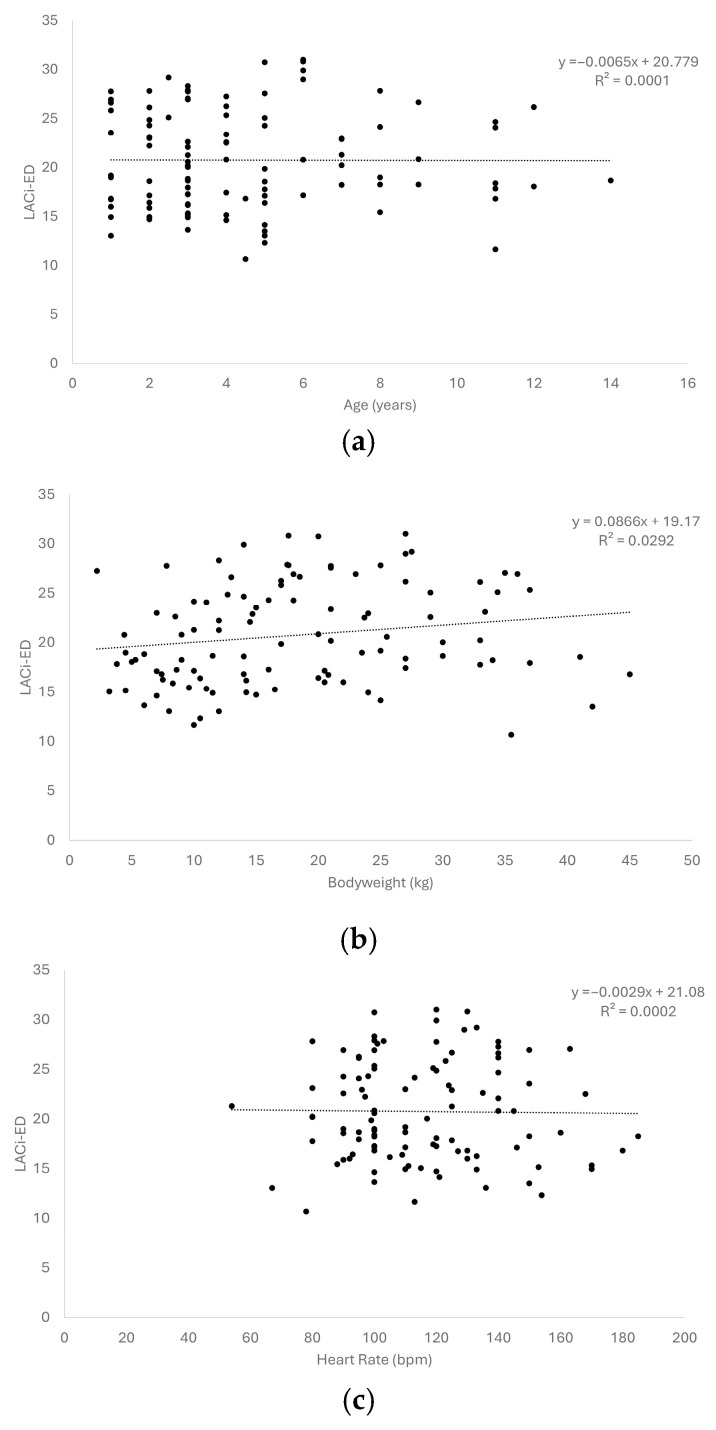
Scatter plots of left atrioventricular coupling index at end-diastole (LACi-ED) values in healthy dogs showed no relationship with age (**a**), bodyweight (**b**), and heart rate (**c**).

We failed to observe any linear relationship between the LACi (both LACi-ED and LACi-ES) and age, bodyweight, or heart rate in healthy dogs (R^2^ < 0.03 for all variables; Figure 2).

Reference values for the LACi-ED and LACi-ES were established using data from the 105 healthy dogs included in the study. For the LACi-ED, reference values ranged from 12.09% to 30.76%, and for the LACi-ES, they ranged from 34.88% to 115.34% (Table 2).

**Figure 3:**
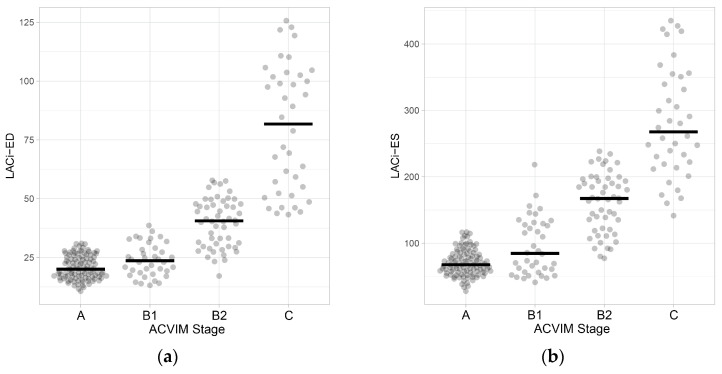
Dot plots of left atrioventricular coupling index values at end-diastole (LACi-ED) (**a**) and at end-systole (LACi-ES) (**b**) in healthy dogs and dogs with myxomatous mitral valve disease at different ACVIM stages. Horizontal lines represent the median values for each group.

**Figure 4:**
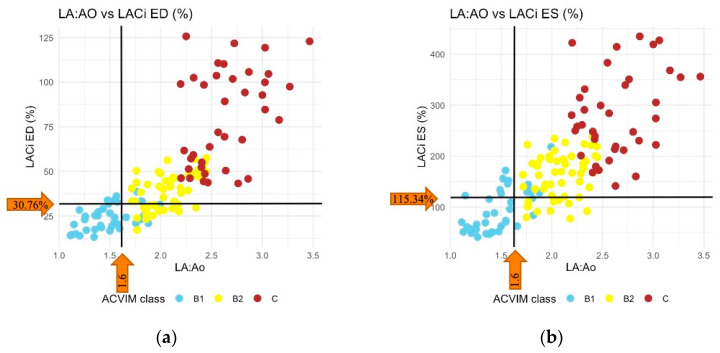
Scatter plot showing classification agreement between left atrioventricular coupling index at end-diastole (LACi-ED) (**a**) or at end-systole (LACi-ES) (**b**) and left atrial-to-aortic-root ratio (LA:Ao). The threshold for identification of left atrial enlargement was 1.6 for LA:Ao, 30.76% for LACi-ED and 115.34% for LACi-ES.

When comparing the LACi values across the different ACVIM stages, both LACi-ED and LACi-ES increased progressively with advancing disease stages (*p* < 0.001 and *p* < 0.02, respectively) (Figure 3 and Figure 4). We found no differences between healthy dogs and those in ACVIM stage B1 for either the LACi-ED or LACi-ES (*p* > 0.20). However, when cutoff values of 30.76% for the LACi-ED and 115.34% for the LACi-ES were applied as reasonable upper working limits for healthy dogs, the LACi-ED and LACi-ES identified left atrioventricular impairment in 9 and 15 stage B1 dogs, respectively (Figure 4). Conversely, LACi-ED and LACi-ES did not identify left atrioventricular impairment in 13 and 12 stage B2 dogs, respectively (Figure 4).

**Figure 5:**
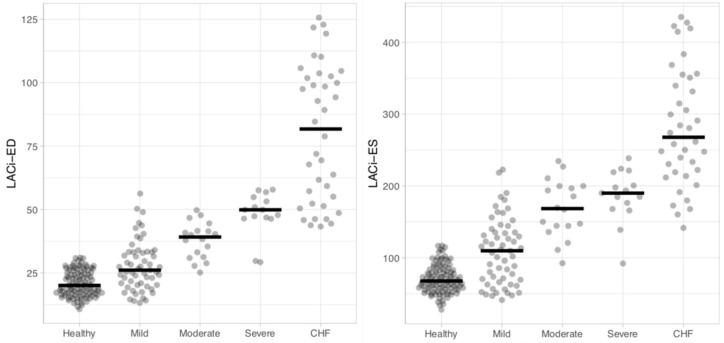
Dot plots of left atrioventricular coupling index values at end-diastole (LACi-ED) and at end-systole (LACi-ES) in healthy dogs, subclinical dogs (mild, moderate and severe) and dogs with congestive heart failure (CHF). Horizontal lines represent the median values for each group.

When comparing the LACi-ED values across the different levels of severity of subclinical MMVD, LACi-ED increased progressively with advancing subclinical disease severity (mild vs. moderate: *p* < 0.001 and moderate vs. severe: *p* = 0.041) (Figure 5). Furthermore, LACi-ED values differed between healthy dogs and dogs with mild subclinical MMVD (*p* < 0.001). Finally, the LACi-ED differed between dogs with severe subclinical disease and dogs with CHF (*p* < 0.001).

**Figure 6:**
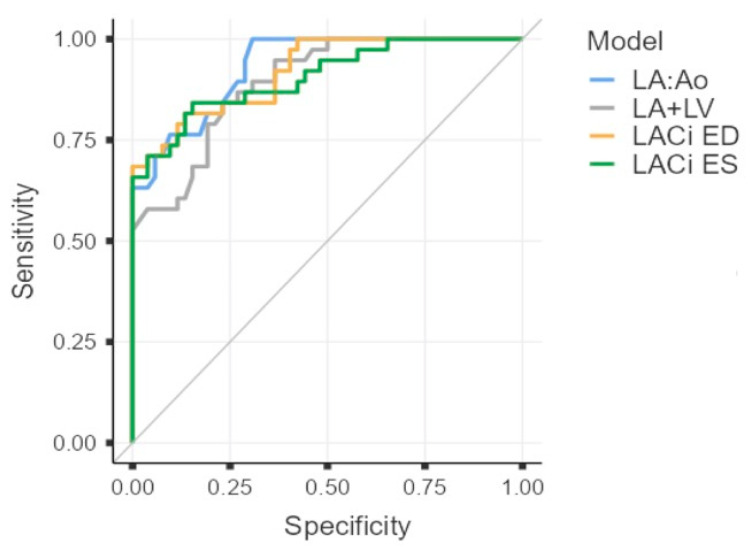
Receiver operating characteristic (ROC) curves for left atrioventricular coupling index at end-diastole (LACi-ED), left atrioventricular coupling index at end-systole (LACi-ES), left atrial-to-aortic-root ratio (LA:Ao) and composite index LA:Ao+LVIDDn (LA+LV) to differentiate dogs with congestive heart failure. The area under the curve (AUC) is 0.920, 0.906, 0.931, and 0.893 for LACi-ED, LACi-ES, LA:Ao, and LA+LV, respectively.

All four indices (LACi-ED, LACi-ES, LA:Ao, and the composite index LA:Ao+LVIDDn) demonstrated similar, good diagnostic accuracy for identifying CHF, with the areas under the curve (AUCs) of 0.920, 0.906, 0.931, and 0.893, respectively (Table 3 and Figure 6). However, the sensitivity and specificity of each method differed (Table 3).

## 4. Discussion

Our study demonstrates that left atrioventricular coupling indices show a progressive impairment of left atrioventricular function in dogs affected by MMVD with advancing ACVIM stages and, within subclinical disease, by advancing disease severity. We describe for the first time the use of the LACi-ED and the LACi-ES as indices of left atrioventricular dysfunction in dogs, providing reference values for these echocardiographic indices in healthy adult dogs. Whether these indices can provide useful prognostic information for dogs with subclinical disease remains to be determined.

In humans, LA and LV volumes used to calculate the LACi are measured at the end-diastole, where the atrium and ventricle are directly connected through the mitral valve, thereby reflecting the interaction between atrial and ventricular function [6]. The LACi has emerged as a promising echocardiographic index for patient prognostication in several clinical scenarios and is expressed as a percentage, with higher values indicating a relatively larger atrial volume compared with ventricular volume, representing a more abnormal atrioventricular coupling state [4,5,6,7]. In our study, we obtained LACi measurements at the end-diastole (LACi-ED) and end-systole (LACi-ES), as these phases represent moments in the cardiac cycle when the LA and LV are directly connected through the mitral valve, and their function and filling pressures are tightly coupled. Both LACi variables showed a significant, progressive increase with advancing disease severity, and their diagnostic accuracy in identifying dogs with CHF was comparable. Therefore, we speculate that left atrioventricular coupling is similarly impaired at the end-diastole and end-systole in dogs affected by MMVD.

Both LACi variables were found to be independent of body weight, age, and heart rate in healthy dogs. Our results differ from those of previous human studies regarding the association with age [6,12]. However, the healthy dogs included in our study had a limited age distribution (median age of four years); therefore, we cannot exclude age-associated cardiac changes in healthy geriatric dogs.

The moderate relationship between LACi-ED and E peak velocity reflects the pathophysiological link between LA enlargement, elevated LA pressure and the early transmitral pressure gradient. We speculate that this relationship is only moderate because early ventricular filling is also influenced by LV relaxation and compliance. The weak relationship with the E/A ratio can be explained by the fact that this parameter is additionally affected by atrial contractile function and HR, making it less directly dependent on LA size alone. The lack of relationship with the LACi-ES indicates that minimal LA volume, which primarily reflects atrial emptying function rather than filling pressure, does not directly influence early transmitral flow.

The LACi values did not differ between healthy dogs and dogs with stage B1 MMVD, suggesting that atrioventricular uncoupling is not yet evident during the early, asymptomatic phase of the disease. Furthermore, a small number of stage B1 dogs showed mildly increased LACi values, which might indicate early impairment of atrioventricular coupling. We hypothesize that these dogs can exhibit subtle left atrial dimensional alterations while still fulfilling the diagnostic criteria for ACVIM stage B1, namely the absence of left ventricular remodelling. This is supported by our observations that LACi-ED values differed between healthy dogs and dogs with mild MMVD (which includes both stage B1 and some stage B2 dogs).

Dogs with stage B2 MMVD showed substantial heterogeneity in LACi values: some exhibited measurements within the normal reference range, whereas others exceeded the established thresholds for healthy dogs. This underscores the lack of clinical utility with the current ACVIM staging scheme. Consequently, we used a newly proposed classification scheme to classify dogs with subclinical disease into three levels of severity: “mild”, “moderate”, and “severe” [11]. When examined using this scheme, the LACi-ED differed between all levels of subclinical disease severity, as well as between healthy dogs and dogs with mild disease, and dogs with severe subclinical disease and those with CHF. We did not apply the scheme to the LACi-ES, because the scheme relies on end-diastolic measurements. Increased LACi values in stage B2 dogs might reflect early atrioventricular uncoupling, suggesting that the dynamic coordination between atrial and ventricular function is beginning to deteriorate even before the onset of clinical signs of CHF. Conversely, normal LACi values can indicate preserved atrioventricular coupling. This heterogeneity suggests that stage B2 encompasses a broad spectrum of structural and functional cardiac changes that are not fully captured by conventional ACVIM-defined staging criteria. From a pathophysiological perspective, an increase in the LACi implies disproportionate left atrial enlargement relative to ventricular size, pointing to impaired left atrial reservoir function and increased atrial pressures. Whether this pattern can serve as an early marker of diastolic dysfunction or left atrial afterload mismatch, and whether this subpopulation is at higher risk of progression to CHF, remain undetermined and warrant further investigation. Overall, these findings highlight the potential prognostic value of the LACi for improved clinical stratification of dogs with stage B2 MMVD.

Left atrioventricular coupling index (LACi) values were markedly elevated in dogs at ACVIM stage C, consistent with the progressive nature of MMVD, which results in chronic volume overload and enlargement of the LA and LV. These findings suggest advanced atrial and ventricular remodelling, as well as marked impairment of atrioventricular coupling. Compared with traditional echocardiographic indices such as the LA:Ao, LVIDDn, and the combined LA:Ao + LVIDDn, the LACi demonstrated comparable diagnostic accuracy for identifying CHF. Specifically, the LACi showed lower sensitivity but higher specificity than the LA:Ao. Although the LACi can represent an additional tool in the echocardiographic assessment of MMVD, particularly for evaluating atrioventricular function and refining risk stratification, further prospective studies are needed to confirm its clinical utility.

Our study has several limitations. As with all retrospective studies, we were unable to control data collection when cases were examined. Moreover, this limited our ability to assess the prognostic value of the LACi over time. In addition, dogs in the MMVD group, particularly those in ACVIM stages B2 and C, were receiving cardiac medications (e.g., diuretics, ACE inhibitors, and pimobendan). These treatments can influence cardiac loading conditions and myocardial function, potentially affecting atrioventricular function and, consequently, LACi values. Furthermore, volumetric measurements were not validated using independent modalities, such as three-dimensional echocardiography or cardiac magnetic resonance imaging, but were previously compared to other methods and validated as a reasonable means of calculating volumes. The two populations (healthy dogs and dogs with MMVD) were not fully comparable in baseline characteristics and clinical status. These differences could influence the LACi values and should be considered when interpreting the results. Finally, intra- and inter-observer variability for LA and LV volume measurements were not assessed in this study, as these parameters have been extensively evaluated in previous studies [13,14,15,16,17,18].

## 5. Conclusions

This study is the first to evaluate left atrioventricular coupling in healthy dogs and in dogs affected by MMVD. The LACi is a novel, non-invasive, and easily obtainable echocardiographic index that reflects the functional relationship between the LA and LV. Our findings indicate a progressive impairment of left atrioventricular function in dogs with MMVD as ACVIM stage advances, and suggest that the LACi might be a useful additional echocardiographic parameter for assessing disease severity and refining risk stratification.

## Figures and Tables

**Table 1:** Clinical and echocardiographic data for 233 dogs included in the study.

	Healthy Dogs	Dogs with MMVD
Number of dogs (male)	105 (51)	128 (59)
Age (years)	4 (1–14)	12 (5–17)
Body weight (kg)	17 (3.8–45)	9.6 (2.5–32.2)
Heart rate (bpm)	11 (54–185)	127 (63–200)
LA:Ao	1.3 (1–1.7)	2.1 (1.1–3.4)
LVIDDn	1.41 (1.10–1.78)	1.83 (1.26–2.55)
LAEDV (mL)	8.97 (1.03–25.20)	13.83 (2.57–131.50)
LAESV (mL)	16.70 (1.80–40.97)	24.78 (4.77–203.47)
LVEDV (mL)	45 (4.03–118.47)	35.40 (7.03–126.83)
LVESV (mL)	35.71 (11.54–82.83)	40.1 (9.27–126.83)
FS (%)	36.2 (26.8–77.4)	42.1 (26.9–70.6)
LACi-ED (%)	20.03 (10.68–31.01)	40.59 (13.17–125.68)
LACi-ES (%)	67.68 (27.81–116.83)	167.40 (41.17–435.07)

Data are presented as median (range) for age, body weight, heart rate, LA:Ao, LVIDDn, LAEDV, LAESV, LVEDV, LVESV and LACi variables. LA, left atrium; Ao, aorta; LVIDDn, left ventricular internal diameter in diastole normalized to body weight; LAEDV, left atrial end-diastolic volume; LAESV, left atrial end-systolic volume; LVEDV, left ventricular end-diastolic volume; LVESV, left ventricular end-systolic volume; FS, fractional shortening; LACi-ED, left atrioventricular coupling index at the end-diastole; LACi-ES, left atrioventricular coupling index at the end-systole.

**Table 2:** Reference intervals for LACi variables in 105 clinically healthy dogs.

Variable	Lower Limit (90% CI ^1^)	Upper Limit (90% CI)
LACi-ED	12.09% (CI 10.68–13.44)	30.76% (CI 28.99–31.01)
LACi-ES	34.88% (CI 27.81–42.07)	115.34% (CI 106.62–116.83)

^1^ CI = confidence interval.

**Table 3:** Receiver operating characteristic curve analysis of left atrioventricular coupling index at end-diastole (LACi-ED), left atrioventricular coupling index at end-systole (LACi-ES), left atrial-to-aortic-root ratio (LA:Ao) and composite index LA:Ao+LVIDDn (LA+LV) for identifying congestive heart failure in dogs affected by myxomatous mitral valve disease.

Variable	Threshold Value	Sensitivity (95% CI ^1^)	Specificity (95% CI)	Area Under the Curve
LACi-ED	58.5	68 (51–82)	100 (93–100)	0.920
LACi-ES	200.8	84 (69–94)	84 (72–93)	0.906
LA:Ao	2.2	100 (91–100)	69 (55–81)	0.931
LA+LV	4.2	84 (69–94)	77 (63–87)	0.893

^1^ CI = confidence interval.

## Data Availability

The original contributions presented in this study are included in the article. Further inquiries can be directed to the corresponding authors.
